# The brain-bone marrow axis and its implications for chronic traumatic brain injury

**DOI:** 10.21203/rs.3.rs-3356007/v3

**Published:** 2023-10-06

**Authors:** Rodney M. Ritzel, Yun Li, Yun Jiao, Sarah J. Doran, Niaz Khan, Rebecca J. Henry, Kavitha Brunner, David J. Loane, Alan I. Faden, Gregory L. Szeto, Junfang Wu

**Affiliations:** 1 Department of Anesthesiology and Shock, Trauma and Anesthesiology Research (STAR) Center, University of Maryland School of Medicine, Baltimore, Maryland, USA.; 2 Department of Neurology, McGovern Medical School, The University of Texas Health Science Center at Houston, Texas, USA.; 3 Department of Chemical, Biochemical and Environmental Engineering, University of Maryland, Baltimore County, Maryland, USA.

## Abstract

Traumatic brain injury (TBI) causes acute and chronic alterations in systemic immune function which contribute to posttraumatic neuroinflammation and neurodegeneration. However, how TBI affects bone marrow (BM) hematopoietic stem/progenitor cells chronically and to what extent such changes may negatively impact innate immunity and neurological function has not been examined. To further understand the role of BM cell derivatives on TBI outcome, we generated BM chimeric mice by transplanting BM from chronically injured or sham congenic donor mice into otherwise healthy, age-matched, irradiated hosts. After 8 weeks of reconstitution, peripheral myeloid cells from TBI→WT mice showed significantly higher oxidative stress levels and reduced phagocytic activity. At eight months after reconstitution, TBI→WT chimeric mice were leukopenic, with continued alterations in phagocytosis and oxidative stress responses, as well as persistent neurological deficits. Gene expression analysis revealed BM-driven changes in neuroinflammation and neuropathology after 8 weeks and 8 months of reconstitution, respectively. Chimeric mice subjected to TBI showed that longer reconstitution periods were associated with increased microgliosis and leukocyte infiltration. Thus, TBI causes chronic activation and progressive dysfunction of the BM stem/progenitor cell pool, which drives long-term deficits in innate immunity and neurological function, as well as altered sensitivity to subsequent brain injury.

## Introduction

Traumatic brain injury (TBI) causes marked changes in both the systemic and central immune system. Neuroinflammation is a major contributor to secondary brain injury and contributes to disruption of the blood-brain barrier (BBB), neurodegeneration and neurological dysfunction (1–3). Importantly, TBI also triggers a systemic immune/inflammatory response. In patients with TBI, the numbers of circulating myeloid cells increase markedly during the first several days after hospitalization and correlate with poor outcomes (4, 5). These myeloid cells are also recruited to the brain where they may contribute to worse outcome and/or neurological recovery (6–9), depending on underlying health conditions, temporal dynamics, and leukocyte composition. These increased levels of circulating immune cells may reflect activation of the hematopoietic system at its most upstream point. Hematopoietic stem/progenitor cells reside at the apex of the hematopoietic hierarchy, continuously giving rise to all cells of the blood and immune system. In response to sterile injury, the marrow rapidly adapts to produce specific cell types that are in high demand via a process known as stress-induced hematopoiesis. As the site of leukocyte production, bone marrow cells serve as critical regulators of neuroimmune interactions in health and disease (10).

In contrast to the acceleration of pro-inflammatory activity in the brain during the acute stages of post-traumatic injury, systemic immune function is broadly suppressed, resulting in an immunocompromised state that can lead to infection. Corroborating evidence indicates that TBI patients are at a substantially heightened risk of nosocomial infection during the first week of hospitalization (11–14). Secondary infection following acute TBI can worsen recovery, lengthen hospital stays, and increase mortality risk (15, 16). The resolution of this early immune/inflammatory response facilitates the recovery phase, however, alterations in peripheral immune composition and function persist throughout life, just as microglial activation may persist indefinitely after TBI. Indeed, our lab and others have experimentally shown that peripheral infections during acute and chronic stages of TBI are more severe compared to sham controls and often cause bidirectional damage (17–22). Although we have demonstrated that chronic microglial activation can aggravate post-traumatic neurodegeneration (23–26), the long-term consequences and progression of bone marrow dysfunction following brain injury remains largely undocumented.

We tested the hypothesis that moderate-to-severe TBI induces inherent changes to the bone marrow compartment that can, in turn, impact chronic immune dysfunction and worsen long-term neurological outcomes. To address this issue, we generated bone marrow chimeric mice using a novel transplantation strategy in which congenic sham and chronic TBI donors were used to reconstitute the immune system of otherwise healthy WT mice. This experimental approach allowed us to untangle the pathological effects of bone marrow cells from that imposed by the brain injury environment, to better understand the contribution of these cells on the development of long-term sequelae following TBI.

## Methods

### Animals and controlled cortical impact model of TBI

Adult male C57BL/6 mice (8–10 weeks, 20–25g) and Pep Boy mice (B6.SJL-Ptprc^a^ Pepc^b^/BoyJ, common name: B6 CD45.1, Strain#: 002014) were obtained from the Jackson Laboratory and housed in the UMB animal facility on a 12-hour light/dark cycle with food and water provided for them *ad libitum*. Controlled cortical impact (CCI) was performed with the TBI-0310 impactor (Precision Systems & Instrumentation, VA) as described in prior studies (23, 27, 28). Following induction of anesthetization with isoflurane, a 10-mm midline incision was made over the skull and a 4-mm craniotomy was made on the central aspect of the left parietal bone of mice under surgical anesthesia. A moderate-severe contusion injury was induced with a 3.5-mm diameter tip at velocity of 4.0 m/s and tissue deformation depth of 1.2 mm. For sham surgery groups, mice were subjected to the same procedures, but didn’t receive a craniotomy and contusion impact.

### Generation of bone marrow chimeras

5–15 minutes prior to irradiation, animals are anesthetized with Ketamine (80–150 mg/kg)/Xylazine (10–16 mg/kg) dissolved in saline. The anesthetic solution was administered intraperitoneally (0.1–0.5 cc) using a 1 m L syringe and 27-gauge needle. Total body irradiation was delivered using a fractionated two dose protocol of 550 rad three to four hours apart (1.22–1.28 Gy/min) to deplete BM prior to transplant (29). Animals were exposed to radiation using a 320kV orthovoltage machine (XRAD 320, Precision X-Ray). Radiation was delivered to the whole body without head shielding. Mice were given prophylactic antibiotics (0.01% Enrofloxacin (Baytril) in their drinking water beginning two days before BM transplantation for up to six weeks. Femur bone marrow cells were harvested from 90d TBI and sham congenic Pepboy (CD45.1) donor mice, and 100 uL of BM cells (1 × 10^6^ cells/mouse) were intravenously injected by retroorbital injection in recipient WT (CD45.2) C57BL/6 mice. Mice were allowed to reconstitute for 8 weeks and 8 months following transplantation.

### Flow cytometry

Whole blood was isolated by external cardiac puncture using EDTA-coated needles. Mice were then perfused with 40 mL of cold saline. 200 μl of blood was lysed for 10 min on ice using red blood cell lysis buffer (Biolegend, Cat# 420301) and then washed with FACS buffer. This was repeated up to three times until sufficient lysis was achieved. Bone marrow was harvested from the ipsilateral femur by flushing with Roswell Park Memorial Institute (RPMI) (Lonza Group, Basel, Switzerland) medium using hydrostatic pressure. The ipsilateral (i.e., craniotomy-side) brain hemisphere was isolated(28). The olfactory bulb and cerebellum were removed, brains were halved along the interhemispheric fissure, and the ipsilateral hemisphere was placed separately in complete Roswell Park Memorial Institute (RPMI) 1640 (Invitrogen, Cat# 22400105) medium and mechanically and enzymatically digested in collagenase/dispase (1 mg/ml, Roche Diagnostics, Cat# 10269638,001), papain (5U/ml, Worthington Biochemical, Cat# LS003119), 0.5 M EDTA (1:1000, Invitrogen, Cat# 15575020), and DNAse I (10 mg/ml, Roche Diagnostics, Cat# 10104159001) for 1 h at 37 °C on a shaking incubator (200 rpm). The cell suspension was washed twice with RPMI, filtered through a 70-μm filter, and RPMI was added to a final volume of 5 mL/hemisphere and kept on ice. Cells were then transferred into FACS tubes and washed with FACS buffer. Blood, bone marrow, and brain cells were then incubated with TruStain FcX Block (Biolegend, Cat# 101320), for 10 min on ice, and stained for the following surface antigens: CD45-eF450 (eBioscience, Cat# 48–0451–82), CD11b-APCeF780 (eBioscience, Cat# 47–0112–82), Ly6C-AF700 (Biolegend, Cat# 128024), and Ly6G-PE (Biolegend, Cat# 127607). The fixable viability dye Zombie Aqua was used for live/dead discrimination (Biolegend, Cat# 423102). Cells were then washed in FACS buffer, fixed in 2% paraformaldehyde for 10 min, and washed once more prior to adding 500 μl FACS buffer. Intracellular staining for Ki67-PECy7 (Biolegend, Cat# 652426) and PCNA-AF647 (Biolegend, Cat# 307912) was performed using Cytofix/Cytoperm Fixation/Permeabilization Kit (BD Biosciences, Cat# 554714) according to manufacturer’s instructions and as described previously(23). Cytokine staining for TNF-PECy7 (Biolegend, Cat# 506324) and IL-1β-PerCPeF710 (eBioscience, Cat# 46–7114–82) was performed after 3h incubation with Brefeldin A (Biolegend, Cat# 420601) at 37 °C followed by fixation/permeabilization. For phagocytic activity, circulating and bone marrow-derived neutrophils were incubated with 0.5–1.0 μm latex carboxylate-modified fluorescent red beads (1:500, Sigma-Aldrich, Cat# L3280). For ROS detection, bone marrow-derived Ly6G^+^ neutrophils were incubated with DHR 123 (5 mM; Life Technologies/Invitrogen, Cat# D23806), cell permeable fluorogenic probes. Cells were loaded for 20 min at 37°C, washed three times with FACS buffer (without NaAz), and then stained for surface markers including viability stain.

Data were acquired on a BD LSRFortessa cytometer using FACSDiva 6.0 (BD Biosciences) and analyzed using FlowJo (Treestar Inc.). At least 5–10 million events were collected for each sample. CountBright Absolute Counting Beads (ThermoFisher, Cat# C36950) were used to estimate cell counts per the manufacturer’s instructions. Data were expressed as counts/hemisphere. Leukocytes were first gated using a splenocyte reference (SSC-A vs FSC-A). Singlets were gated (FSC-H vs FSC-W), and live cells were gated based on Zombie Aqua exclusion (SSC-A vs Zombie Aqua-Bv510). Bone marrow LSK+ cells were identified as lineage^−^c-Kit^+^Sca1^+^ (Biolegend, Cat# 133307, 405226, 105837, and 108111). Blood and splenic neutrophils were identified as Resident microglia were identified as CD45^+^CD11b^+^Ly6G^+^. CD45^int^ CD11b^+^Ly6C^−^ population, whereas peripheral leukocytes were identified as CD45^hi^CD11b^+^ myeloid cells or CD45^hi^CD11b^−^ lymphocytes. Cell type–matched fluorescence minus one (FMO) controls were used to determine the positivity of each antibody.

### Behavioral testing

#### Y-maze

To assess spatial working memory, Y-maze spontaneous alternation test was performed at indicated time with the same protocols described in prior publications (23, 27). During the test, mice were randomly placed in one of three identical arms (A, B, C) that consisted of the Y-maze (Stoelting Co.) and allowed to freely move within the maze for 5 min. Arm entries were recorded and percentage of alternation was calculated with the following equation: total alterations × 100/(total arm entries − 2). For indications of spatial working memory, the mouse must score significantly higher than 50%.

#### Spontaneous motor activity

Locomotor activity was measured with the open field (OF) test for spontaneous movement (23, 30). At the start of the test, each mouse was individually placed in a corner of the open field arena (22.5 cm × 22.5 cm) facing towards the wall. The mouse was allowed to explore the chamber for 5 min and movement parameters were recorded with the Any-Maze software (Stoelting Co.). Total distance travelled, average speed, time mobile vs. immobile and rearing/elongation behavior were indicative of motor activity levels.

#### Tail-suspension test

For assessment of depression-like behavior, tail-suspension test was performed (23, 30, 31). Based on the observation that mice would develop an immobile posture when placed in an inescapable hemodynamic stress of being hung by their tail, each mouse was suspended at a height of 28 cm using 3M adhesive tape. The tail tip was prevented from wrapping around the rod while being suspended. During the 5-minute testing period, the total time of immobility was recorded, which is defined as passive hanging and complete motionlessness. Foam padding (3” thick) was placed under the suspension beam to avoid harm in case of a fall during the experiment.

#### Grip strength

Grip strength was measured with a digital grip strength meter (Bioseb BP, In Vivo Research Instruments, France) as previously described (23, 28). In brief, forelimb grip strength of both paws was measured together by placing the mouse on a mesh wire grid attached to the device’s force transducer. Once a secure grip was confirmed, the mouse was then held by its tail and slowly pulled away from the bar. The maximal average force exerted on the mesh wire grid was recorded and an average number of 10 trials per day was obtained for each mouse. The final grip force was normalized by body weight before comparison between groups.

#### Rotarod

Locomotor function was assessed with rotarod as described in previous studies (23, 28). Briefly, the mouse was placed on a rotarod device (IITC Life Science Inc.) with acceleration settings at 4 to 40 rpm over 90 s. The latency to fall was recorded, with a maximum trial time of 300s. Individual scores from three trials for each mouse were averaged to reflect their motor function.

#### Catwalk XT automated gait analysis

For assessment of motor coordination, the CatwalkXT system (Noldus, RRID: SCR_004074) was used for recording and analysis of gait (23, 30). In a dark room, mice were placed on the glass walkway and allowed to traverse from one end to the other. Contact between the paws and glass surface results in illuminated footprints after light reflection. Footprint images were video recorded by a camera positioned under the walkway. Videos from each trial were analyzed with the CatWalk XT 9.1 software program at minimum threshold of 80 (a.u. ranging from 0 to 225). Following identification of footprint, data pertaining to static and dynamic gait parameters were generated for each run. The mean score from 3 consecutive runs (per animal/time points) are analyzed for statistical significance. Runs in which the mouse stopped midway or turned around during a run were excluded from analysis.

### Multiplex Enzyme-Linked Immunosorbent Assay (ELISA)

Multiplex ELISA was performed on a Luminex FlexMAP 3D (Millipore Sigma) using a premixed 32-plex cytokine magnetic bead panel (MilliporeSigma, Cat# MCYTMAG-70K-PX32) following the kit protocol. Undiluted plasma (25 μl) was assayed in technical duplicates. Cytokine concentrations were determined by interpolation to known standard curves. Samples below the limit of detection were set to the lowest standard concentration. Analytes that were not within detectable limits or showed no change are not shown.

### Magnetic isolation of immunolabeled cells

For magnetic isolation of blood neutrophils, red blood cells were lysed as above, and single cell suspensions were washed in selection media. Cells were then incubated with Fc block for 10 min on ice as noted above. Neutrophils were isolated using the MojoSort Mouse Ly6G Selection Kit (Biolegend, Cat# 480058) according to manufacturer’s instructions. For magnetic isolation of femur bone marrow LSK+ cells, single cell suspensions were blocked and stained with biotinylated-lineage cocktail (Biolegend, Cat# 133307) and stem/progenitor cells were negatively selected using streptavidin-conjugated nanobeads (Biolegend, Cat# 480015). Lineage-negative cells were then stained with c-Kit-APC (Biolegend, Cat# 105811) for positive selection with anti-APC-conjugated nanobeads (Biolegend, Cat# 480071). Finally, Lin-cKit+ bone marrow cells were stained with Sca1-APC (Biolegend, Cat# 108111) for positive selection with anti-APC-conjugated nanobeads. The resulting neutrophil and LSK+ cell populations were stored in Qiazol lysis reagent (Qiagen, Cat# 79306) at −80 °C.

### RNA isolation and NanoString assay

Total RNA was isolated from bone marrow and brain tissue samples with the RNeasy Mini Kit (Qiagen, Cat# 74104) according to the manufacturer’s instructions. Isolated RNA was eluted in 40 μl of RNase-free water and tested with an Agilent 2100 Bioanalyzer to ensure it met specifications for purity (RNA integrity number higher than 9). A portion of the total RNA sample (25 ng/μl) was loaded to the Nanostring nCounter platform (Nanostring Technologies, Seattle, WA) to measure transcript counts. The Stem Cell, Neuroinflammation, and Neuropathology panels were used for assessment of bone marrow and brain hemisphere tissue samples respectively. Gene transcript counts of each sample were normalized with NanoString nSolver 4.0 before further downstream analysis in RStudio Version 2022.12.0 + 353 with various bioinformatic packages. Partial least-squares discriminant analysis (PLSDA) was performed with the ropls package in R (32) and graphs were drawn with ggplot2. For heatmap illustrations, graphs were drawn with ComplexHeatmap (33). Pathway enrichment analysis was performed on the Enrichr website from Maayan lab (34, 35). All pairwise comparisons of “A vs. B” should be interpreted as “A relative to B” in text and figures that pertains to fold-change. Volcano plots depicting the fold change and p-value of pairwise comparisons were drawn with the EnhancedVolcano package in R. Genes with a raw p-value of less than or equal to 0.05 were considered as differentially expressed genes (DEGs) and were further analyzed with pathway enrichment analysis. Percentage of genes modified by TBI injury were calculated as number of DEG versus total number of genes tested for that cellular function.

### RNA sequencing of neutrophils

Isolated neutrophils were sent to Novogene (Sacramento, CA) for RNA extraction, mRNA library preparation (poly A enrichment) and sequencing on NovoSeq 6000 PE150 platform. Bioinformatic analyses were performed on High Performance Computing facility taki CPU cluster at University of Maryland Baltimore County. The quality of FastQ data were analyzed using FastQC v0.11.8 (*Available at:* http://www.bioinformatics.babraham.ac.uk/projects/fastqc/). Transcript-level level abundance was quantified using Salmon V1.0.0 (36), mapping to GRCm38 (mm10) mouse reference genome, then converted to gene-level abundance using tximport. Differential expression analysis was performed using DESeq2 (37). Partial least squares-discriminant analysis (PLS-DA) was performed using mixOmics (38). Tximport, DESeq2, and mixOmics packages were run in R v4.1.2 in RStudio v1.2. Sample-level enrichment analysis (SLEA) were performed as described by Gundem *et al.* (https://genomemedicine.biomedcentral.com/articles/10.1186/gm327). Briefly, the null distribution of each sample was calculated as the mean expression of 10,000 sets of genes the same size as the scored gene sets. The mean expression of scored gene sets were calculated and z-scored based on the null distribution (SLEA score). Gene sets were downloaded from Gene Ontology Biological pathway database for Epigenetic regulation of gene expression (GO:0040029) and Chromatin remodeling (GO:0006338).

### Senolytic drug treatment

Aged (18-month-old) male C57BL/6 mice (N=10/group) from Charles River were administered Navitoclax (ABT-263, Selleck Chemicals, Cat# S1001), a potent inhibitor of Bcl-2, by oral gavage daily at 12:00 for two weeks. ABT-263 was dissolved in vehicle (10% ethanol, 30% PEG 400, 60% Phosal50) as described previously (39). Mice were weighed and given ABT-263 (50 mg/kg body mass) or vehicle control alone by oral gavage. Behavioral testing was performed at baseline (days −7 to −1) prior to drug administration (starting d0), and again after the two-week treatment period. At one-week post-treatment, mice were subject to CCI and examined at 72h post-injury.

### Statistics

Data from individual experiments are presented as means ± SEM. Data were analyzed by Student’s T-test or Mann-Whitney for two group comparisons, one-way analysis of variance (ANOVA) with Tukey or Bonferronni multiple comparisons test, and two-way ANOVA analysis with Tukey’s post hoc correction for multiple comparisons. All behavioral and *ex vivo* studies were performed by an investigator blinded to condition, treatment, and surgical condition. Statistical analysis was performed using GraphPad Prism software v. 9.0 (GraphPad Software, Inc., La Jolla, CA). Outliers were determined using Grubb’s test. *P* ≤ 0.05 was considered statistically significant.

### Study Approval

All surgical procedures and animal experiments were performed according to protocols approved by the University of Maryland School of Medicine Institutional Animal Care and Use Committee (IACUC).

### Data Availability

Bulk RNA-Seq data were deposited in the NCBI’s Gene Expression Omnibus database (pending GEO#). Source data for NanoString analysis have been archived at Zenodo (https://zenodo.org/record/8397111).

## Results

### TBI causes stress-induced myelopoiesis, chronic activation of bone marrow stem/progenitor cells, and long-term bone marrow failure.

**Figure 1.**
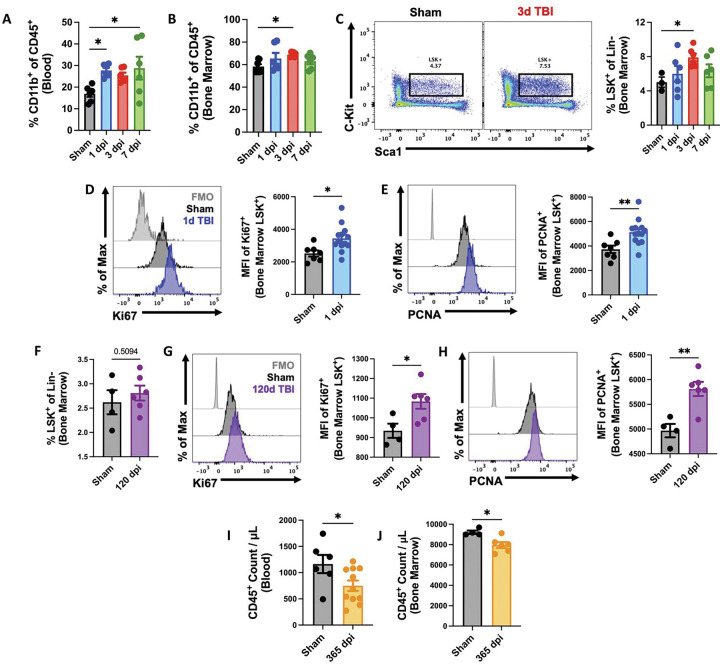
Femur bone marrow responses at acute and chronic stages following TBI. **(A-E)** Flow cytometry was performed in whole blood and femur bone marrow at 1, 3, and 7d post-injury. TBI increased the percentage of myeloid cells (% CD11b+ of CD45+ cells) in blood (**A**) and bone marrow (**B**). Percentages of lineage-c-Kit+Sca1+ (LSK+) stem/progenitor cells were elevated in bone marrow after TBI (**C**). A representative dot plot of LSK+ populations at 3d TBI is indicated in the left panel of C. Quantification of LSK+ cells is shown in the right panel of **C**. The mean fluorescence intensity (MFI) of cell cycle markers Ki-67 (**D**) and PCNA (**E**) were seen significantly increased in the LSK+ population at 1d post-injury. n=6–10 mice/group. **(F-H)** The proliferative status of LSK+ cells was assessed at 120 days post-injury (dpi). n=4 (Sham) and 6 (TBI) mice/group. **(I-J)** Chronic TBI at 365 dpi caused a significant reduction in circulating (**I**) and bone marrow (**J**)-derived white blood (CD45+) cells. n=6–11(blood) and 4–6 (bone marrow) mice/group. For all histograms, light gray=fluorescence minus one (FMO) control. Data were analyzed using one-way ANOVA group analysis with Tukey’s test for multiple comparisons (**A-C**) or Mann-Whitney for two group comparisons (D-J). **p<0.01, *p<0.05.

Recently we reported that moderate-to-severe TBI caused alterations in the bone marrow compartment that subsequently led to leukopenia and reduction in white blood cell production in the femur (24, 40). To begin our next series of investigations into the brain-bone marrow axis, we examined the effect of TBI on stress-induced myelopoiesis. We found a significant increase in circulating myeloid cells during the first week of injury (Figure 1A), consistent with previous reports of acute neutrophilia (40, 41). This coincided with a significant increase in myeloid cell production in femur bone marrow (Figure 1B). Closer examination of the bone marrow stem cell compartment revealed a statistically insignificant trend toward higher percentages of LSK+ stem/progenitor cells at 72h after TBI (Figure 1C), implying hematopoietic activation at the highest level. Indeed, we found that the LSK+ population exhibited increased protein expression of two markers of actively cycling and proliferating cells, Ki-67 and PCNA (Figure 1D–E), consistent with the *de novo* production of myeloid cells following TBI. To assess the persistence of stem cell/progenitor activation after TBI, we evaluated the proliferative status of LSK+ cells at 120 days post-injury (dpi). Although no changes in the frequency of LSK+ cells were seen, TBI caused a continued elevation in Ki-67 and PCNA expression relative to sham controls (Figure 1F–H). As expected, we also observed a significant reduction in circulating and bone marrow-derived white blood cells at 365 dpi (Figure 1I–J), consistent with replicative senescence. Taken together, our findings suggest that TBI induces chronic activation of the bone marrow compartment which eventually leads to decreased production and output of leukocytes that could significantly increase risk of infection.

### TBI induces chronic transcriptomic alterations in bone marrow stem/progenitor cells and blood neutrophils.

**Figure 2.**
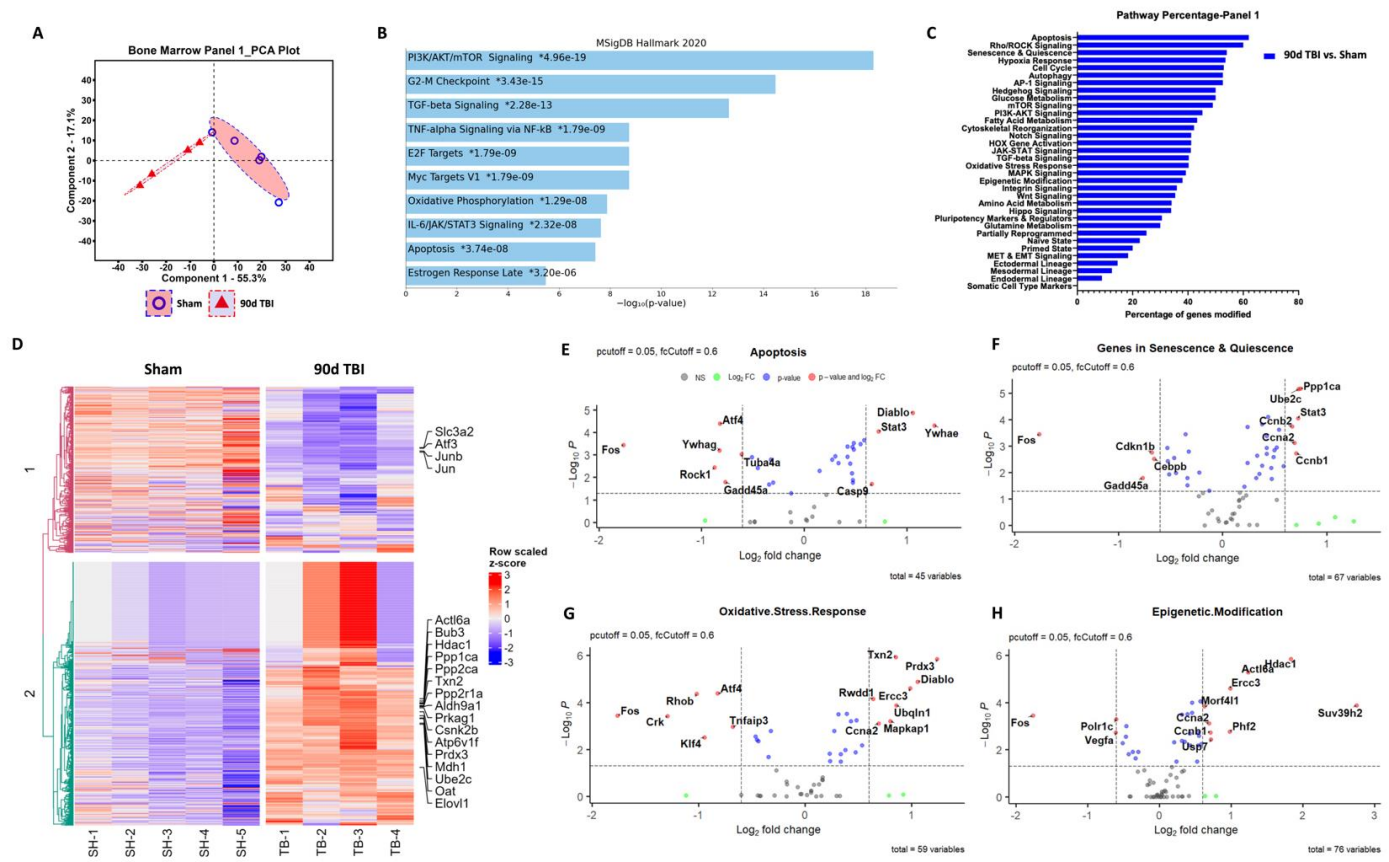
TBI causes long-term alterations in femur bone marrow stem/progenitor cells. The transcriptomic profile of femur bone marrow lineage-c-Kit+Sca1+ (LSK+) cells at 90 days post-injury (dpi) was assessed using the NanoString nCounter Stem Cell panel. **(A)** Principal component analysis (PCA) plot indicated that the two main principal components of variation were captured on the x- and y-axis, respectively, showing a clear separation of clusters between the Sham and TBI groups. **(B)** Pathway enrichment analysis with the MSigDB Hallmark 2020 database revealed that several gene networks related to hematopoietic stem cell proliferation and self-renewal, inflammatory activation, and senescence-associated were enriched after chronic TBI. **(C)** Pathway analysis based on gene annotations given by NanoString revealed high percentage of genes related to Apoptosis, Rho/ROCK signaling, Senescence & Quiescence, Hypoxia Response, and Cell Cycle pathways being modified by TBI. **(D)** Heatmap of genes that are uniquely altered in the most differentially expressed genes between Sham and 90 dpi. Color coding was based on z-score scaling. **(E-H)** Volcano plot analyses for the Apoptosis (**E**), Senescence and Quiescence (**F**), Oxidative Stress Response (**G**), and Epigenetic Modification (**H**) pathways showed the most differentially and significantly expressed genes within each network. n=4–5 mice/group.

To confirm the chronic activation of the bone marrow compartment after TBI we evaluated the transcriptomic profile of femur LSK+ cells at 90 dpi using the NanoString nCounter Stem Cell panel. PCA of all normalized gene counts revealed a distinct separation of sham and TBI samples on the first principal component accounting for 55.3% of total variance (Figure 2A). Pathway enrichment analysis with the MSigDB Hallmark 2020 database revealed that several gene networks involved in hematopoietic stem cell proliferation and self-renewal (i.e., PI3K/AKT/mTOR signaling: *Eif4e, Ptpn11, Cdkn1b, Ppp1ca*), inflammatory activation (i.e., TGF-b: *Tgfb1, Ifngr2, Ppp1ca, Ctnnb1* IL-6/JAK/STAT3: *Tgfb1, Ifngr, Jun, Il6st, Cd44, Grb2* and TNF/NFkB signaling: *Ifngr2, Id2, Gadd45a, Jun, Il6st, Cd44*), and other biological processes (i.e., Myc targets VI, Oxidative Phosphorylation, and Apoptosis signaling) were enriched after chronic TBI (Figure 2B). To further validate these findings, we performed an additional pathway analysis using gene annotations provided by NanoString for this panel. This analysis identified an increased percentage of genes modified in Apoptosis, Rho/ROCK signaling, Senescence & Quiescence, Hypoxia Response, and Cell Cycle pathways (Figure 2C). A heatmap displaying the most differentially expressed genes in each group show TBI-mediated decreases in *Junb* and increases in *Bub3*, *Hdac1*, and *Prdx3*, for example (Figure 2D). Volcano plot analyses for the Apoptosis (Figure 2E), Senescence and Quiescence (Figure 2F), Oxidative Stress Response (Figure 2G), and Epigenetic Modification (Figure 2H) pathways show the most differentially and significantly expressed genes within each network. Of note, genes encoding cell cycle progression (i.e., *Ccnb2*, *CCna2*, and *Ccnb1*) and DNA damage-responsive proteins such as p53 and Rad1 were significantly up-regulated at late timepoints following TBI. Taken together, moderate-to-severe TBI causes long-term alterations in distally located bone marrow stem/progenitor cells that affect transcriptional networks important for regulating apoptosis, cell cycle progression, inflammatory signaling, and senescence and quiescence.

**Figure 3.**
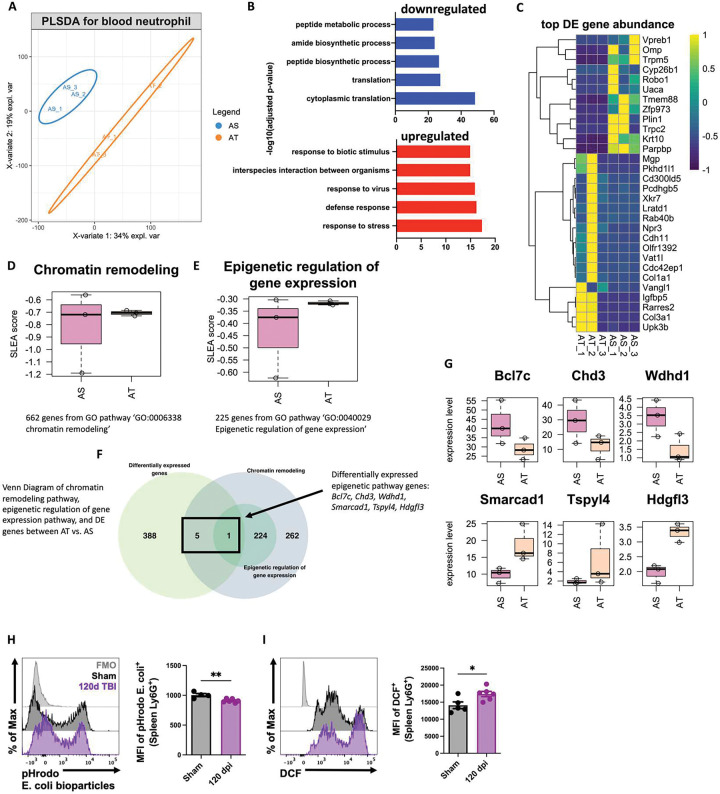
Blood neutrophil functions are chronically dysregulated during the chronic stages of TBI. Bulk RNA-seq was performed on blood neutrophils at 120 dpi. **(A)** PLSDA of all normalized gene counts revealed a clear separation of aged sham and aged TBI samples into individual groups across the first two principal components. n=3 mice/group. **(B)** Pathways involved in biosynthetic processes and translation were down-regulated after TBI, whereas those involved in stress and viral defense responses were up-regulated. **(C)** Heatmap of unsupervised clustering of the top 30 differentially expressed genes. Color coding was based on z-score scaling. **(D-E)** Sample-level enrichment analysis (SLEA) for chromatin remodeling pathway (**D**) and epigenetic regulation (**E**) of gene expression. **(F)** Venn Diagram of chromatin remodeling pathway, epigenetic regulation of gene expression pathway, and differentially expressed (DE) epigenetic pathway genes genes between AT vs. AS. **(G)** Six out of 268 genes from chromatin remodeling pathway were differentially expressed after TBI, including Smarcad1, Tspyl4 and Hgfl3 up-regulated after TBI and Bcl7c, Chd3 and Wdhd1 down-regulated after TBI. **(H-I)** Neutrophil function including phagocytosis (MFI of pHrodo E. coli., **H**) and oxidative stress levels [mean fluorescence intensity (MFI) of DCF, **I**] from the spleen was evaluated by flow cytometry. n=4–6 mice/group. For all histograms, light gray=fluorescence minus one (FMO) control. Data were analyzed using Mann-Whitney for two group comparisons. **p<0.01, *p<0.05. AS: aged sham; AT: aged TBI.

Next, we examined neutrophil function during the chronic stages of TBI (120 dpi) using bulk RNA-seq. PLSDA of all normalized gene counts revealed a clear separation of sham and TBI samples into individual groups across the first two principal components (Figure 3A). We found that pathways involved in biosynthetic processes and translation were down-regulated after TBI, whereas those involved in stress and viral defense responses were up-regulated (Figure 3B). Unsupervised clustering of the top 30 differentially expressed genes identified *Parpbp*, *Krt10*, *Zfp973*, *Tmem88*, and *Trpm5* as being down-regulated following TBI (Figure 3C). Given the relatively short life cycle of neutrophils we also wanted to determine whether TBI chronically altered genes involved in epigenetic modifications. To confirm this, we performed sample-level enrichment analysis (SLEA) for chromatin remodeling pathway and epigenetic regulation of gene expression (Figure 3D–E). Epigenetic regulation of gene expression pathway was altered in neutrophils from the chronic TBI group. Additionally, 6 out of 268 genes from chromatin remodeling pathway were differentially expressed after TBI, including Smarcad1, *Tspyl4* and *Hgfl3* up-regulated after TBI and *Bcl7c, Chd3* and *Wdhd1* down-regulated after TBI (Figure 3F–G). Finally, to better understand the chronic effects of TBI on neutrophil function flow cytometry was applied. TBI-induced changes in spleen neutrophils were observed with regard to phagocytosis and oxidative stress levels, consistent with our previous work(40) (Figure 3H–I). These findings suggest that chronic TBI promotes chromatin remodeling in circulating neutrophils which may have downstream effects on innate immunity during the chronic phase of TBI.

### Immune systems derived from chronic TBI donor mice exhibit impaired innate immune function associated with neurological deficits.

**Figure 4.**
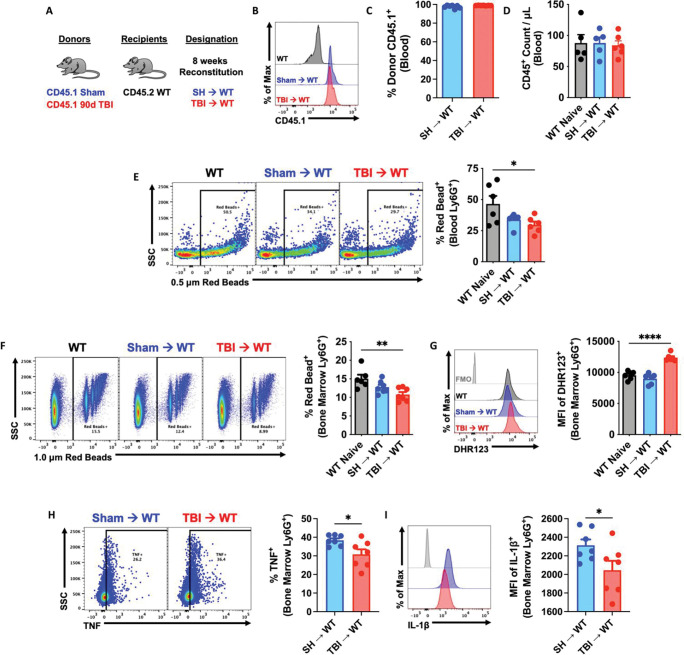
Bone marrow cells transplanted from chronic TBI mice reprogram host innate immune function in the absence of the brain injury environment. **(A)** Using a novel bone marrow transplantation approach, sham and 90 days post-injury (dpi) donor cells were transplanted into irradiated, congenic WT recipients. Femur bone marrow cells were harvested from 90 dpi and sham congenic Pepboy (CD45.1) donor mice, and 100 ml of BM cells (1 × 10^6^ cells/mouse) were intravenously injected by retroorbital injection in recipient WT (CD45.1) C57BL/6 mice. Mice were allowed to reconstitute for 8 weeks following transplantation. **(B-D)** Blood donor cells (CD45.1, **B-C**) and leukocyte (CD45+, **D**) were counted using flow cytometry. n=5–6 mice/group. **(E-G)** Circulating and bone marrow-derived Ly6G+ neutrophils from TBI→WT chimeric mice exhibited reductions in 0.5–1.0 mm Red Beads+ cells and increase in the mean fluorescence intensity (MFI) of DHR123+ ROS. Left panels of **E-F** are representative dot plots. n=5–6 mice/group. **(H-I)** Bone marrow-derived Ly6G+ neutrophils from TBI→WT chimeric mice showed significant reductions in TNF and IL-1b production. Left panels of **H** are representative dot plots of TNF+ cells. n=7 mice/group. For all histograms, light gray=fluorescence minus one (FMO) control. Data were analyzed using one-way ANOVA group analysis with Tukey’s test for multiple comparisons (**E-G**) or Student’s T-test for two group comparisons (**H-I**). ****P<0.0001, **p<0.01, *p<0.05. SH: Sham, WT wildtype.

Our findings indicated that TBI causes persistent alterations in bone marrow cells. We postulated that the chronic effects of TBI on innate immunity, as described here and elsewhere (42), are cell-intrinsic and can be recapitulated in otherwise healthy mice using a novel bone marrow transplantation approach wherein sham and 90 dpi donor cells are transplanted into irradiated, congenic WT recipients (Figure 4A). After 8 weeks of reconstitution, ~99% of host cells were donor-derived and no differences in blood leukocyte counts were seen, compared to non-irradiated controls (Figure 4B–D). Circulating and bone marrow-derived neutrophils from TBI→WT chimeric mice exhibited reductions in phagocytic activity (Figure 4E–F) that coincided with increased oxidative stress levels (Figure 4G), consistent with our earlier findings(40). Furthermore, significant reductions in TNF and IL1β production were also seen TBI→WT neutrophils (Figure 4H–I). Together, these results indicate that TBI induces transcriptional alterations in bone marrow cells that subsequently and inherently reprogram innate immune function, even in the absence of the brain injury environment.

**Figure 5.**
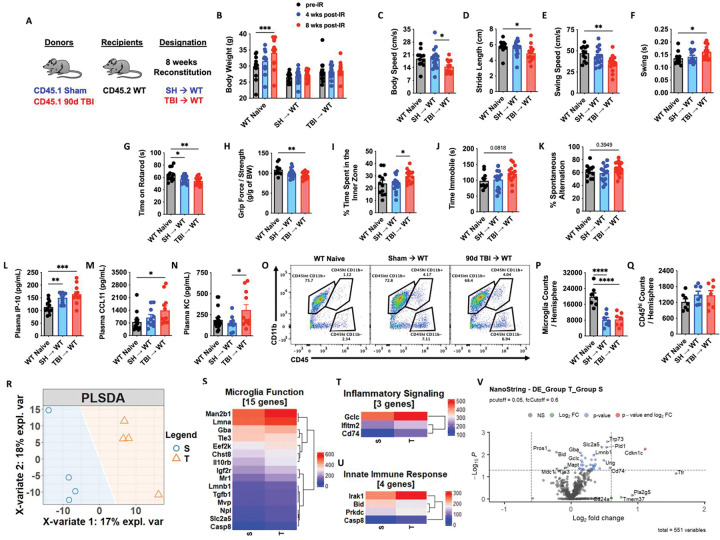
Short-term reconstitution of bone marrow cells transplanted from chronic TBI mice affect host neurological function and neuroinflammatory signaling. **(A)** Chimera paradigm is illustrated. Mice were allowed to reconstitute for 8 weeks following transplantation. n=11–16 mice/group. **(B)** Body weight of animal was monitored before and at 4 and 8 weeks (wks) after irradiation (IR). **(C-F)** Graphs showed parameters (body speed, stride length, swing speed, and swing durations) of CatWalk gait analysis tested at 8 weeks after IR. **(G-J)** Locomotor function and depressive-like behavior were assessed in the accelerating rotarod (**G**), Grip strength (**H**), and Open field (**I**), and tail suspension (**J**) tests. **(K)** Short-term spatial working memory was evaluated in Y-maze test. **(L-N)** Plasma cytokine levels were examined using multiplex Enzyme-Linked Immunosorbent Assay (ELISA). The pro-aging/neurodegenerative cytokines IP-10, CCL11, and KC were significantly increased in TBI→WT mice compared to controls. n=10–13 mice/group. **(O-Q)** Brain microglia and CD45^hi^ leukocytes were detected using flow cytometry. A representative dot plot of leukocyte populations in the brain is illustrated in **O**. Quantification of CD45^int^CD11b^+^ microglia (**P**) and CD45^hi^CD11b^+^ myeloid cells (**Q**) are shown. **(R-V)** The transcriptomic profile of brain tissue at 8 weeks after IR was assessed using the NanoString nCounter Neuroinflammation panel. n=4 mice/group. The Partial least squares-discriminant analysis (PLSDA) is illustrated (**R**). Heatmap of genes that are associated with Microglial Function (**S**), Inflammatory Signaling (**T**), and the Innate Immune Response (**U**) are shown. Color coding was based on Average Transcription Counts (normalized by housekeeping genes). Volcano plot analysis is indicated (**V**). Data were analyzed using one-way ANOVA group analysis with Tukey’s test for multiple comparisons. W: WT Naive; S: SH→WT; T: TBI→WT. ****P<0.0001, ***p<0.001, **p<0.01, *p<0.05. SH: Sham.

Thus far we have demonstrated that TBI can profoundly alter the hematopoietic compartment, however, its bidirectional impact on the brain warranted further attention. Employing the same chimera paradigm as before (Figure 5A), we confirmed no differences in body weight between SH→WT and TBI→WT groups following irradiation (Figure 5B). A battery of neurobehavioral tests was performed to assess motor, mood, and cognition. Interestingly, TBI→WT chimeric mice exhibited significantly poorer gait performance as evidenced by decreases in body speed, stride length, and swing speed (Figure 5C–E). Consistent with the latter finding, swing durations were also increased in TBI→WT mice compared to non-irradiated controls (Figure 5F). The latency to fall from an accelerating rotarod was significantly decreased in both chimeric groups (Figure 5G), however, TBI→WT mice displayed significantly decreased forelimb grip strength (Figure 5H). Whereas TBI→WT mice spent significantly more time in the center area of the open field chamber compared to SH→WT mice (Figure 5I), no statistical differences were found for the tail suspension test (depressive-like behavior) (Figure 5J). Nor were there differences in short-term spatial working memory, as reflected by the Y-maze test (Figure 5K). Together, these results indicate that the chronic alterations observed in bone marrow cells following TBI can affect neurological function independent of the brain injury environment.

To further validate these findings, we next examined plasma cytokine levels and gene signatures of neuroinflammation in the chimeric mice. Increased plasma concentrations of the pro-aging/neurodegenerative cytokines IP-10, CCL11, and KC were more significantly altered in TBI→WT mice compared to controls (Figure 5L–N). Surprisingly, despite reduced numbers of microglia in both irradiated groups (Figure 5O–P), no differences in the number of peripherally-derivedCD45^hi^ leukocytes were detected in the brain by flow cytometry (Figure 5Q). The NanoString nCounter Neuroinflammation gene panel revealed distinct separation of the non-irradiated WT group and two irradiated chimeric groups in the PLSDA plot, with loose clustering of SH→WT and TBI→WT groups (Fig. 5R). However, a deeper analysis revealed several genes associated with Microglial Function (Figure 5S), Inflammatory Signaling (Figure 5T), and the Innate Immune Response (Figure 5U) were differentially expressed in TBI→WT compared SH→WT mice. Volcano plot analysis showed several genes that were significantly upregulated in the cortex of TBI→WT mice relative to SH→WT mice, including *Cdkn1c* (p57) and *Cd74* (Figure 5V). Collectively, these results suggest that inherent changes to the bone marrow compartment affect neurological function and are associated with systemic inflammatory mediators and imbalances in neuroinflammatory signaling.

### Aging post-transplantation causes bone marrow failure and neurological dysfunction in mice reconstituted with chronic TBI bone marrow cells.

**Figure 6.**
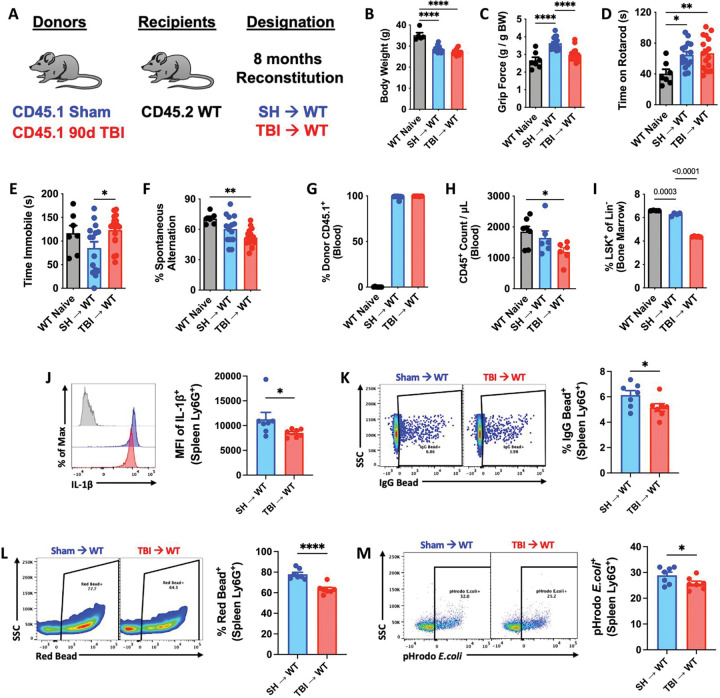
Long-term reconstitution of bone marrow cells transplanted from chronic TBI mice causes innate immune dysfunction and neurological decline in host mice. **(A)** Chimera paradigm is illustrated. Mice were allowed to reconstitute for 8 months following transplantation. **(B)** Body weight was monitored at 8 months after irradiation (IR). **(C-F)** A battery of neurobehavioral tests was performed to assess forelimb grip strength (**C**), time on rotarod (**D**), time immobility in the tail suspension test (**E**), and cognitive function in Y-maze test (**F**). **(G-H)** Blood donor cells (CD45.1, **G**) and leukocyte (CD45+, **H**) were counted using flow cytometry. **(I)** Percentages of bone marrow LSK+ frequencies were detected. **(J)** Spleen Ly6G+ neutrophils were examining using flow cytometry, showing reduced mean fluorescence intensity (MFI) of IL-1b+ cells in TBI→WT mice compared to SH→WT animals. Light gray=fluorescence minus one (FMO) control. **(K-M)** Phagocytic engulfment of IgG-coated beads (**K**), latex beads (**L**), and pHrodo-labeled E.coli particles (**M**) were detected in spleen Ly6G+ neutrophils. Left panels of **K-M** are representative dot plots. Data were analyzed using one-way ANOVA group analysis with Tukey’s test for multiple comparisons (**B-I**) or Student’s T-test for two group comparisons (**J-M**). n=7 mice/group. ****P<0.0001, **p<0.01, *p<0.05. SH: Sham.

Because TBI is a chronic neurodegenerative disease with gradually evolving and progressive pathology, we hypothesized that the effects of aging and time post-injury would further worsen the TBI-like phenotype recapitulated in the TBI→WT chimeric mice. To address this question, we allowed the irradiated hosts to reconstitute for 8 months after transplantation (Figure 6A). The continuous, long-term hematopoietic supply and replenishment of bone marrow-derived cells from 90 dpi donors did not chronically alter host body weights compared to SH→WT mice (Figure 6B), eliminating this confounding variable from our behavioral analysis. TBI→WT mice displayed significantly poorer forelimb grip strength compared to SH→WT mice (Figure 6C), but no change in time spent on an accelerating rotarod (Figure 6D). However, TBI→WT mice exhibited significantly increased depressive-like behavior and cognitive deficits on the tail suspension and Y-maze tests, respectively (Figure 6E–F). These findings imply that bone marrow cells, independently, can contribute to long-term functional deficits, including memory impairment, following TBI.

Further analysis of the immune system showed high donor reconstitution rates of both chimeric groups (Figure 6G), with significantly fewer circulating white blood cells and LSK+ frequencies in TBI→WT mice (Figure 6H–I). Consistent with our earlier results, neutrophils showed a sustained decrease in IL1β production in TBI→WT mice (Figure 6J), and impaired phagocytic engulfment of IgG-coated beads, latex beads, and pHrodo-labeled *E.coli* particles (Figure 6K–M). These findings suggest that increased time post-injury results in a persistent and progressive worsening in innate immune function, indicative of bone marrow failure.

**Figure 7.**
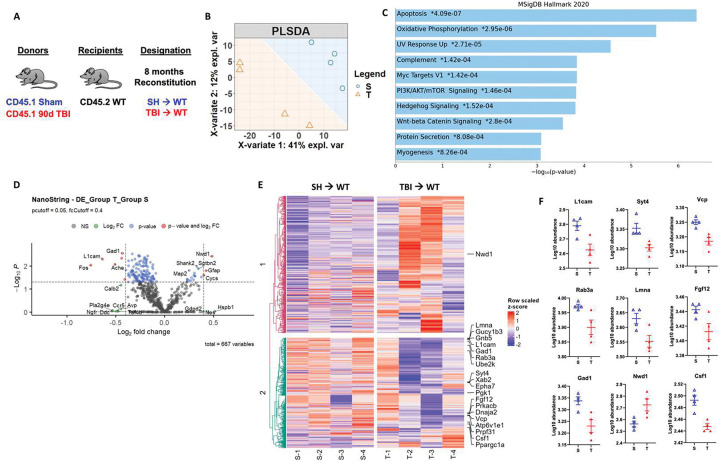
Long-term reconstitution of bone marrow cells transplanted from chronic TBI mice worsens ongoing neurodegeneration at the transcriptomic level in host brains. The transcriptomic profile of whole brain hemisphere tissue at 8 months after irradiation was assessed using the NanoString nCounter Neuropathology panel. **(A)** Chimera paradigm is illustrated. **(B)** Partial least squares-discriminant analysis (PLSDA) plot showed a separation of clusters between the TBI→WT (T) and SH→WT (S) groups. **(C)** Pathway enrichment analysis with the MSigDB Hallmark 2020 database revealed that several gene networks related to apoptosis, oxidative phosphorylation, UV response, and complement pathways were enriched in TBI→WT compared to SH→WT mice. **(D)** Volcano plot analyses are shown between two groups. **(E)** Heat map analysis of the top 20 most differentially expressed genes are indicated. Color coding was based on z-score scaling. **(F)** Normalized transcription count of the top 9 genes presented as Log10 abundance. n=4 mice/group.

Given that long-term reconstitution of otherwise healthy WT mice with chronic TBI donor bone marrow stem cells resulted in neurological dysfunction, we investigated whether injury-induced alterations in bone marrow cells might have neurodegenerative potential. Because the chimeric mice were not head-shielded during irradiation, the resulting DNA damage and neuronal loss provided us with a unique neurodegenerative model system in which to study the neuromodulatory effects of the bone marrow compartment. Following the same 8-month reconstitution paradigm with sham and 90 dpi donor mice (Figure 7A), we performed NanoString analysis on cortex/hippocampal tissue using the nCounter Neuropathology gene panel. Separation between SH→WT and TBI→WT groups was apparent in the PLSDA plot (Figure 7B). We found significant enrichment in genes involved in the apoptosis, oxidative phosphorylation, UV response, and complement pathways in TBI→WT compared to SH→WT mice (Figure 7C). Volcano plot analysis revealed significant increases in *Gfap* and reductions in *L1cam, Gad1,* and *Fos* in TBI→WT relative to SH→WT mice, consistent with astrogliosis and concomitant neuronal loss or dysfunction (Figure 7D). Heat map analysis of the top 20 most differentially expressed genes further highlight comparative reductions in *Fgf12* and *Csf1*, two growth factors important for maintaining neuronal and microglial homeostasis (Figure 7E–F). Moreover, we also found decreases in neuron-related genes such as *Lmna*, *Syt4*, *Rab3a*, and *Vcp* in the cortex/hippocampus of TBI→WT mice. Taken together, our transcriptomic findings support the notion that brain injury-induced changes in the bone marrow compartment can independently drive and progressively worsen ongoing neurodegeneration.

### Chimeric mice subjected to TBI show age-dependent microgliosis and leukocyte infiltration.

**Figure 8.**
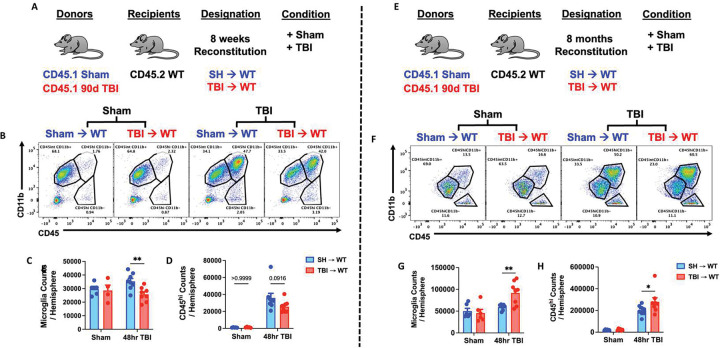
The acute neuroinflammatory response to a subsequent TBI is sensitized with increasing time post-injury. **(A)** Chimera paradigm is illustrated. Mice reconstituted for 8 weeks following transplantation were subjected to TBI. **(B)** Representative dot plots of immune populations in the ipsilateral brain hemisphere at 48 h after TBI. **(C-D)** Quantification of CD45^int^CD11b^+^ microglia and CD45^hi^CD11b^+^-infiltrating myeloid cell counts per hemisphere are shown for Sham and TBI in chimeric mice. n=4–8 mice/group. **(E)** Chimeric mice reconstituted for 8 months following transplantation were subjected to TBI. **(F**) A representative dot plot of leukocyte populations in the ipsilateral brain at 48 h after TBI. **(G-H)** Quantification of CD45^int^CD11b^+^ microglia (**G**) and CD45^hi^CD11b^+^ myeloid cells (**H**) are shown. n=6–8 mice/group. Data were analyzed using two-way ANOVA group analysis with Tukey’s test for multiple comparisons. **p<0.01, *p<0.05. SH: Sham.

To better understand the chronicity of TBI-induced bone marrow dysfunction and its dynamic regulation of brain function, we evaluated and contrasted the acute neuroinflammatory response to a subsequent TBI in 8-week and 8-month chimeric mice reconstituted with sham and 90 dpi donor cells (Figure 8A). Shorter time post-injury (90 dpi + 8 weeks reconstitution) was associated with a significant decrease in microgliosis and a downward trend in bone marrow-derived CD45^hi^ leukocyte infiltration compared to SH→WT controls (Figure 8B–C). Longer time post-injury (90 dpi + 8 months reconstitution), however, was associated with a significant increase in microgliosis and CD45^hi^ leukocyte infiltration (Figure 8D–E). These findings suggest there may be an acute refractory period followed by a progressively increased sensitivity to a repeated TBI which is mediated by bone marrow cells.

### The senolytic agent ABT-263 improves behavioral performance of aged mice at baseline but does not attenuate neuroinflammation in the acutely injured brain.

**Figure 9.**
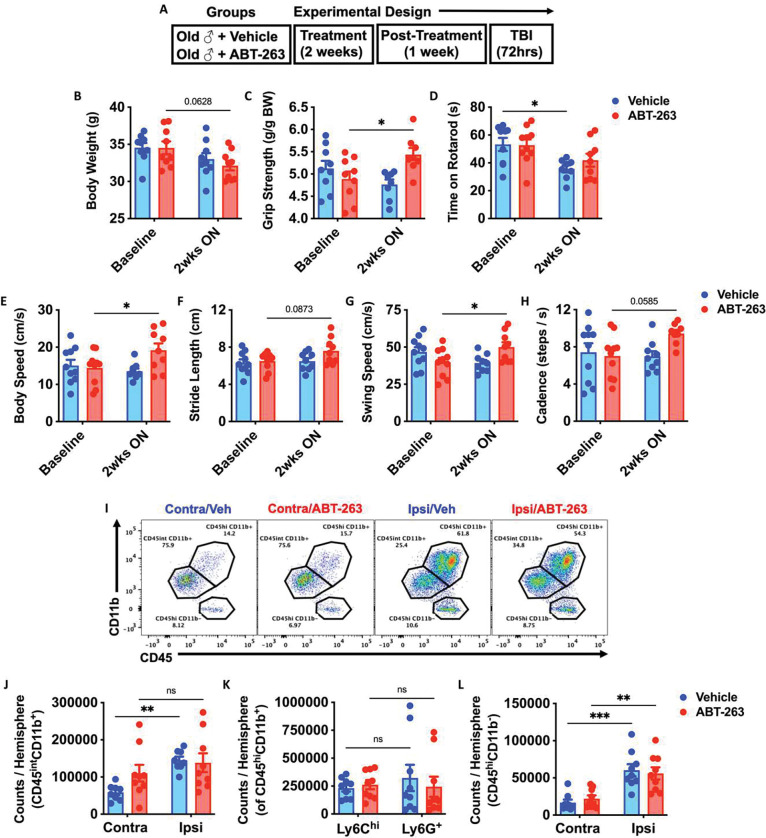
The senolytic drug, ABT-263, has beneficial effects on normal age-related motor function but does not confer robust protection to acute TBI. **(A)** Experimental design. Naïve aged mice (18-month-old) were treated with ABT-263 (50 mg/kg) or vehicle (Veh) once daily for 2 weeks by oral gavage. After two-weeks on and one week off drug, mice were subjected to TBI up to 72h post-injury. **(B)** Body weight was monitored before and at 2 weeks (wks) after the treatment. **(C-H)** A battery of neurobehavioral tests was performed to assess forelimb grip strength (**C**), time on rotarod (**D**), and CatWalk gait analysis for body speed (**E**), stride length (**F**), swing speed (**G**), and cadence (**H**). **(I)** Representative dot plots of immune populations in the ipsilateral (Ipsi) and contralateral (Contra) brain hemisphere at 72 h after TBI. **(J-L)** Quantification of CD45^int^CD11b^+^ microglia, CD45^hi^CD11b^+^-infiltrating myeloid cell, CD45^hi^CD11b^−^-infiltrating lymphocytes counts per hemisphere are shown for Sham, TBI, treatment in aged mice. n=9–10 mice/group. Data were analyzed using two-way ANOVA group analysis with Tukey’s test for multiple comparisons. ***p<0.001, **p<0.01, *p<0.05. SH: Sham.

Finally, our results implicated TBI-induced bone marrow senescence as a potential driver of neurodegeneration and neurological decline. To address the therapeutic potential of senolytic treatment, we prophylactically treated aged (18-month-old) male WT mice with the Navitoclax (ABT-263), which has been experimentally shown to rejuvenate the aging hematopoietic niche (39). Naïve aged mice were treated with ABT-263 once daily for 2 weeks by oral gavage (Figure 9A). No statistically significant change in body weight from baseline was seen in either group (Figure 9B). In line with previous studies(39, 43), we observed significant age-related improvements in forelimb grip strength after ABT-263 treatment (Figure 9C). Moreover, ABT-263 prevented worsening in rotarod performance weeks later (Figure 9D), and markedly improved gait performance from baseline compared to the vehicle control group (Figure 9E–H). Surprisingly, however, ABT-263 treatment did not confer protection to acute TBI, as evidenced by equivalent numbers of microglia and infiltrating bone marrow-derived CD45^hi^ leukocytes (Figure 9I–L). Collectively, these data suggest that the senolytic drug, ABT-263, has beneficial effects on normal age-related motor function, but do not appear to protect against acute TBI in aged animals.

## Discussion

Although there has been increased recognition of the impact of TBI on systemic organ systems, chronic posttraumatic immunological changes have been less well studied, particularly with regard to bone marrow-derived immune cells. Our findings demonstrate that experimental TBI causes chronic activation of the bone marrow, characterized by acute myelopoiesis that is followed by the late emergence of senescence cellular signatures and subsequent failure to produce white blood cells. Reconstituting the immune system of otherwise healthy mice with bone marrow from chronic TBI mice caused innate immune dysfunction and neurological impairment. The nature and degree of changes were dependent on the length of time post-injury. Our data suggest that bone marrow cells undergo cellular reprogramming that appears to be stably propagated by stem/progenitor cells to downstream hematopoietic derivatives, impacting the composition and function of the systemic immune system (40). Importantly, we also show that bone marrow cell alterations following TBI may play a role in promoting neuroinflammation and associated neurological dysfunction. Moreover, after trauma, bone marrow cells appear sensitized to subsequent brain injuries.

Isolating the effects of central and peripheral immunity on TBI outcomes is difficult and not without limitation. The caveats of generating bone marrow chimeric mice in this study include exposure to irradiation and inability to decipher the contribution of specific leukocyte subtypes. In contrast, we argue that not head-shielding provided a low-level neurodegenerative response in the brain that could be further modified in either direction by bone marrow-derived cells. In addition, while the 8wk reconstitution period could be influenced by a pleotropic effect of longer-lived bone marrow cells and short-term hemopoietic stem/progenitor cells, the 8mos reconstitution allowed sufficient time for self-renewal of long-term hematopoietic stem cells and, therefore, was more likely indicative of maladaptive epigenetic imprinting of the descendent immune system.

We and others have previously demonstrated that experimental TBI causes long-term changes in immune function, which increase infection susceptibility and worsen outcomes; these effects are part of broader bi-directional brain-systemic interactions after injury that senstitizes the brain to subsequent challenges (17–22). Ritzel *et al.* demonstrated that moderate-to-severe TBI causes chronic alterations in the peripheral immune system that are reminiscent of accelerated aging (40). Acute stress-induce myelopoiesis was accompanied by neutrophilia and peripheral immune suppression during the early phase. Deficits in phagocytosis and elevated oxidative stress levels persisted for months after injury, associated with the development of leukopenia and bone marrow failure at one-year post-injury (24, 44). Importantly, we found that bone marrow-derived macrophage cultures harvested from chronic (90d) TBI mice exhibited significantly higher sensitivity to LPS stimulation compared to macrophages cultured from sham mice (45, 46). This is consistent with the concept of “trained immunity”, a functional state of the innate immune system that is characterized by long-term epigenetic and metabolic reprogramming of cells associated with potent immune responses (47). These results suggested that TBI causes inherent changes in bone marrow myeloid progenitors that are potentially transmissible and pathogenic. Such impairment in phagocytosis and ROS production in neutrophils were recapitulated in our novel bone marrow chimera model. Moreover, these immune deficits persisted for up to 8-months post-reconstitution, implying that such changes may persist indefinitely. The development of bone marrow failure at chronic timepoints following TBI was further linked to the induction of hematopoietic stem cell senescence. We examined the possibility that alterations in stem/progenitor cells resulted in downstream alterations in the epigenetic regulation of neutrophils, and identified six epigenetic pathway genes that were differentially regulated by chronic TBI: *Bcl7c* (B-cell CLL/lymphoma 7 protein family member C), *Chd3* (chromodomain helicase DNA binding protein 3), *Wdhd1* (WD repeat and HMG-box DNA binding protein 1), *Smarcad1* (SWI/SNF-related matrix-associated actin-dependent regulator of chromatin subfamily A containing DEAD/H box 1), *Tspyl4* (Testis-specific Y-encoded-like protein 4), and *Hdgfl3* (Hepatoma-Derived Growth Factor, Related Protein 3). Although the specific role of these epigenetic regulators for innate immune memory following TBI remains to be investigated, posttraumatic epigenetic modifications during adult hematopoiesis may contribute to the pathogenic neuroimmune effects found in TBI→WT mice.

It is generally understood that the early infiltration of bone marrow-derived cells into the injured brain can markedly amplify the neuroinflammatory response to TBI (4). The infiltration of peripheral myeloid cells typically waxes and wanes within the first week of injury (48). Beyond these acute timepoints, however, the capacity for distally located bone marrow cells to modulate neuroinflammation in the brain during the chronic phase of TBI has yet to be established. The present study identified several genes that were upregulated in the brain of TBI→WT chimeric mice after an 8wk reconstitution period. These include *CdKn1c* (p57/Kip2), *Lmnb1* (Lamin B1), *CD74*, *Pld1* (Phospholipase D1), *Trp73* (p73), *Ung* (Uracil DNA Glycosylase), and *Slc2a5* (Solute carrier family 2 member 5). CD74 encodes a cell membrane, high-affinity receptor for macrophage migration inhibitory factor (MIF); the latter has recently been shown to promote neurodegeneration and cell death in an experimental model of TBI (49). In addition to genes that regulate the inflammatory response, we identified several genes involved in age-related senescence. For example, p57 and p73 play a critical role in coordinating the cellular response to stress, being able to drive to both apoptosis and cellular senescence (50, 51). Upregulation of Ung, a DNA repair gene, suggests that TBI→WT mice may have had further accelerated irradiation-induced DNA damage response (52), while downregulation of Bid, a pro-apoptotic member of the Bcl-2 protein family, suggests involvement in senescent cell formation (53). With extended time post-injury/reconstitution, we saw further perturbations in apoptotic, UV response, oxidative phosphorylation, and complement pathways that have each been previously implicated in chronic neurodegeneration after TBI (54–59). Taken together, our findings suggest that peripheral bone marrow cells augment chronic neuroinflammatory responses at a distance and may even accelerate cellular senescence in the brain after injury.

The chronic phase of moderate-to-severe TBI is highlighted by neurological decline, including motor disability and cognitive worsening (60, 61). We and others have previously reported long-term neurological deficits in mice using the CCI model (24, 25, 27, 62). In this study, we showed that TBI inherently altered bone marrow cells which could independently drive neurological dysfunction in the absence of primary brain injury. Behavioral deficits were more diverse during the 8wk reconstitution period but persisted at 8 months after reconstitution. It is important to note that we did not observe an increase in CD45^hi^ bone marrow-derived leukocytes in the brain of chimera groups. Our data indicate TBI chronically alters innate immune cell function. Chronic TBI chimeric mice (TBI→WT) showed elevated plasma concentrations of CCL11, a pro-aging chemokine previously demonstrated to impair cognition and neurogenesis (63). TBI-associated increases in KC/CXCL1 may be due to increased oxidative stress in innate immune cells (64), and further may amplify peripheral tissue inflammation by recruiting CXCR2-positive cells (65).

Our chimera also model provided an opportunity to evaluate the effect of a “repeated” moderate-to-severe TBI specifically on bone marrow cells, although host mice were not directly exposed to prior TBI. We observed that time post-injury affects subsequent responses to TBI. Specifically, increased time post-injury sensitized the immune system to respond more vigorously to TBI, as evidenced by increased bone marrow-derived leukocyte infiltration and secondary microgliosis. Bone marrow responses to another TBI were refractory, with shorter time intervals post-injury, suggesting possible epigenetic memory or immune priming of trauma-related stress signals that may be acutely adaptive (66, 67). However, with aging, bone marrow cells may become primed, or rewired for long-lasting trained immunity. Thus, the effects of a single TBI on the bone marrow compartment may decrease or increase sensitivity of these cells to subsequent TBI, depending on the time since the prior injury.

TBI causes premature senescence in numerous cell types (23, 68, 69). Administration of senolytic drugs has proven beneficial in models of brain injury and neurodegenerative disease (39, 43, 69, 70). However, few studies to date have examined senolytic therapies in preclinical TBI studies (69). We selected ABT-263 based on previous reports of its ability to rejuvenate the aging hematopoietic stem cell niche and improve several parameters of aging (39, 43). Although we did not observe a protective effect of pre-treatment on TBI-mediated neuroinflammation in aged mice, we found that the baseline improvements in behavioral performance that are consistent with previous reports (39, 43). Moreover, future studies of senolytic therapies in TBI should evaluate dose ranges, timing and duration of administration, and longer-term follow-up.

In summary, the present study highlights the importance of the brain-bone marrow axis in shaping the outcome trajectory of chronic brain injury as we age. The inextricable link between the immune system and the brain may provide an alternative therapeutic strategy to interfere with or slow neurodegenerative disease progression that is less invasive, more accessible, far reaching, and self-renewing. These findings support the notion that biomarkers of blood leukocyte health reflect brain health and the trajectory of neurological disease.
